# Evaluation of the Role of Traditional Medicare and Medicare Advantage Plan in Annual Wellness Visits

**DOI:** 10.1093/geroni/igaf122.1066

**Published:** 2025-12-31

**Authors:** Zhang Zhang, Nancy Schoenborn, Katherine Miller, Jennifer Wolff, Daniel Polsky

**Affiliations:** Johns Hopkins Bloomberg School of Public Health, Baltimore, Maryland, United States; Johns Hopkins University School of Medicine, Baltimore, Maryland, United States; Johns Hopkins Bloomberg School of Public Health, Baltimore, Maryland, United States; Johns Hopkins University - School of Public Health, Baltimore, Maryland, United States; Johns Hopkins University, Baltimore, Maryland, United States

## Abstract

**Background:**

The Medicare Annual Wellness Visit (AWVs) was introduced in 2011 as a preventive services visit. Less is known about the differential uptake of the AWV by Medicare insurance coverage type - a consequence of the increasing beneficiary enrollment shifts from TM to Medicare Advantage (MA) plans. This study aims to quantify the differential effects of Medicare insurance coverage type (MA versus TM) on AWV uptake for key subpopulations.

**Method:**

We used 20% nationally representative Medicare insurance claims data from 2018-2019. Probit models assessed the likelihood of AWV uptake, with subgroup analyses by age, race/ethnicity, dual eligibility, chronic conditions, and ADRD status. We included 8,799,206 beneficiaries aged 65 and older. The outcome is whether to have an AWV; the independent variable is the Medicare insurance coverage type.

**Result:**

Over 1/3 (37.3%) of beneficiaries received an AWV in 2019. MA enrollees were 4.4 percentage points more likely to receive an AWV than TM enrollees (p < 0.001). Subgroup analysis showed higher AWV uptake in MA across all key subgroups of interest (all p < 0.001), with the largest differences among the oldest-old adults aged above 85 + (5.8 percentage points), dual eligibles (11.8 percentage points), Black beneficiaries (9.0 percentage points), and those with ADRD (6.6 percentage points).

**Conclusion:**

Enrollment in an MA plan is associated with a higher probability of AWV uptake, particularly among vulnerable populations from racial and ethnic minorities, dual eligibility, and those diagnosed with ADRD. These findings highlight MA’s potential role in promoting preventive care and health equity.

